# Discontinuation of Denosumab: Gradual Decrease in Doses Preserves Half of the Bone Mineral Density Gain at the Lumbar Spine

**DOI:** 10.1002/jbm4.10731

**Published:** 2023-05-18

**Authors:** Michel Laroche, Guillaume Couture, Yannick Degboé

**Affiliations:** ^1^ Centre de Rhumatologie CHU Purpan – Université Toulouse III Toulouse cedex France

**Keywords:** BONE MINERAL DENSITY, DENOSUMAB, DISCONTINUATION, OSTEOPOROSIS

## Abstract

Stopping treatment for osteoporosis with denosumab (Dmab) leads to a major and rapid loss in bone mineral density (BMD) and a risk of vertebral fracture. Subsequent treatment with bisphosphonate (Bp) does not completely prevent this bone loss. We carried out a prospective pilot study to find out whether the gradual dose reduction with denosumab could prevent this bone loss. We proposed a therapeutic protocol consisting in reducing the doses of Dmab to women treated with Dmab for postmenopausal osteoporosis. Six months after the last dose of Dmab 60 mg, the subsequent injection was performed with a reduced dose of 30 mg, and the month‐12 injection was a 15‐mg injection. BMD and serum C‐terminal telopeptide of type I collagen (CTX) were measured at the start of treatment with Dmab (T0), at the last dose with 60 mg (T1), and at 6 months (T2) and 12 months (T3) after the last 15 mg Dmab injection. We included 13 patients aged 68.7 ± 3 years, and treated with Dmab for 45.2 ± 5 months. At the lumbar spine, 39% of the initial gain in BMD was preserved 1 year after the last dose (15 mg). Conversely, at the hip, the bone loss at the end of the treatment reduction protocol was equivalent to the initial gain. The mean CTX level was 166 ± 152 pg/mL 6 months after the last dose (T2; 15 mg), and 549 ± 425 pg/mL 12 months after the last dose (T3; 15 mg). One patient presented two vertebral fractures, 8 months after the last dose of Dmab (15 mg). Gradual dose reduction of denosumab (30 mg then 15 mg) does not prevent bone loss in the hip and partially maintains the initial gain at the spine. © 2023 The Authors. *JBMR Plus* published by Wiley Periodicals LLC. on behalf of American Society for Bone and Mineral Research.

## Introduction

The off‐treatment extension studies with denosumab (Dmab) have shown a rebound in bone turnover markers and a parallel loss in bone mineral density (BMD) following discontinuation of Dmab.^(^ ^1^ ^)^ Consequently, multiple vertebral fractures have been reported after Dmab discontinuation.^(^ ^2^, ^3^ ^)^ Recently, Sølling and colleagues^(^ ^4^ ^)^ used zoledronate (ZOL) in 59 patients treated for 4.6 ± 1.6 years with denosumab to prevent this bone loss. The effect was incomplete: whatever the mode of administration of ZOL, ie, 6 or 9 months after stopping Dmab or when the markers of bone turnover increased, bone loss was, respectively, −4.8%, −4.1%, and −4.7% 12 months after stopping Dmab, at the spine. Other studies have confirmed the partial effect of ZOL or other bisphosphonates.^(^ ^5^, ^6^, ^7^ ^)^

Could a gradual decrease in Dmab doses mitigate the rebound phenomenon of bone remodeling and attenuate bone loss? We proposed a Dmab therapeutic dose‐reduction protocol in 13 patients treated for postmenopausal osteoporosis.

Our objective was to find out whether a gradual reduction in the dose of Dmab reduces the rebound phenomenon and bone loss caused by the discontinuation of this drug.

## Patients and Methods

### Patients

A total of 120 with postmenopausal osteoporosis have been treated with Dmab in our center since the product was marketed. We studied the files of these patients and reconvened in consultation those whose treatment by Dmab had been prescribed for at least 36 months. We performed a dual‐energy X‐ray absorptiometry (DXA) scan on these patients. We included women with postmenopausal osteoporosis, treated for at least 3 years of Dmab, without bisphosphonate treatment prior to Dmab, with a *T*‐score >−2.5 standard deviation (SD) at the two sites (spine, total hip) at inclusion, and without osteoporotic fracture over the last 2 years. Twenty patients met the inclusion criteria and 13 accepted our protocol.

### Ethics

This pilot, monocentric, observational study was approved by the Toulouse II CCP. All patients signed informed consents.

### Treatment regimen

**Fig. 1:**
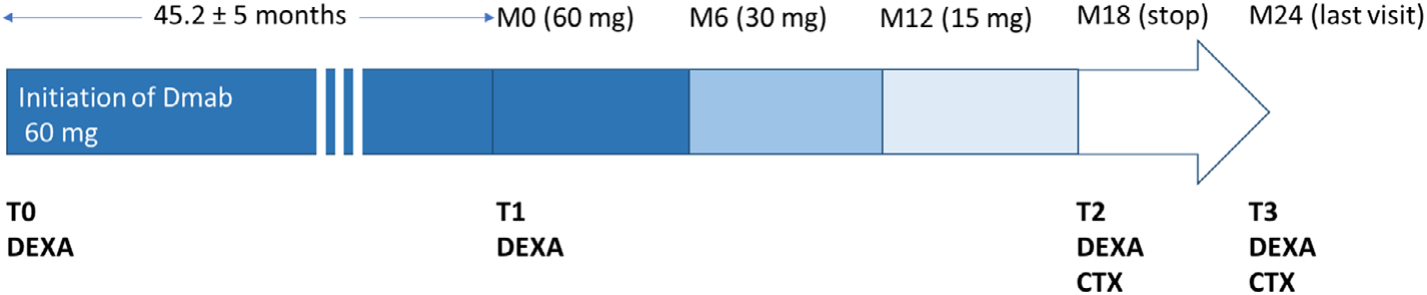
Overview of the study. CTX = crosslaps measurement; Dmab = denosumab. T0: denosumab initiation; T1: last 60 mg denosumab injection; T2: 6 months after; the 15 mg injection of denosumab; T3: 12 months after the 15 mg injection of denosumab.

Six months after the last dose of Dmab 60 mg, they received an injection of 30 mg, followed 6 months later by an injection of 15 mg (Fig. 1). The treatment injection was performed in a standardized way, by nurses from our outpatient clinic, using a graduated syringe to deliver half of the content (30 mg) of the commercial vial, and then a quarter of the content (15 mg).

### Bone assessment

BMD was measured at the spine (L_2_–L_4_) and total hip sites using DXA (Lunar Prodigy; GE Healthcare, Cardiff, UK) by a single experimenter, at the onset of treatment with Dmab (T0), upon discontinuation (T1), 6 months (T2) and 12 months (T3) after the 15 mg injection.

Serum crossLaps (C‐terminal telopeptide of type I collagen [CTX]) measurements were performed at T2 and T3 (Fig. 1). (IDS‐iSYS immunoassay system using chemiluminescent detection).

### Statistical analysis

Data was expressed as mean ± SD. Variation of bone mineral was expressed as a percentage of the initial values. We tested whether values had a Gaussian distribution (Shapiro‐Wilk test). Multiple groups comparison was performed by a one‐way ANOVA and Tukey's multiple comparison test (Gaussian distribution). Correlation between bone loss and CTX variation was assessed by Pearson correlation test (Gaussian distribution). A *p* value <0.05 was considered statistically significant, with a 95% confidence interval (CI).

## Results

### Characteristics of patients at inclusion

The mean age of our population was 68.8 ± 5 years. Regarding prevalent fractures: three patients had a history of vertebral fracture; two patients had wrist fracture. The mean lumbar *T*‐score was −2.3 ± 0.1 and the mean total hip *T*‐score was −2.2 ± 01. The mean duration of denosumab treatment was 45.2 ± 5 months.

### Evolution

**Fig. 2:**
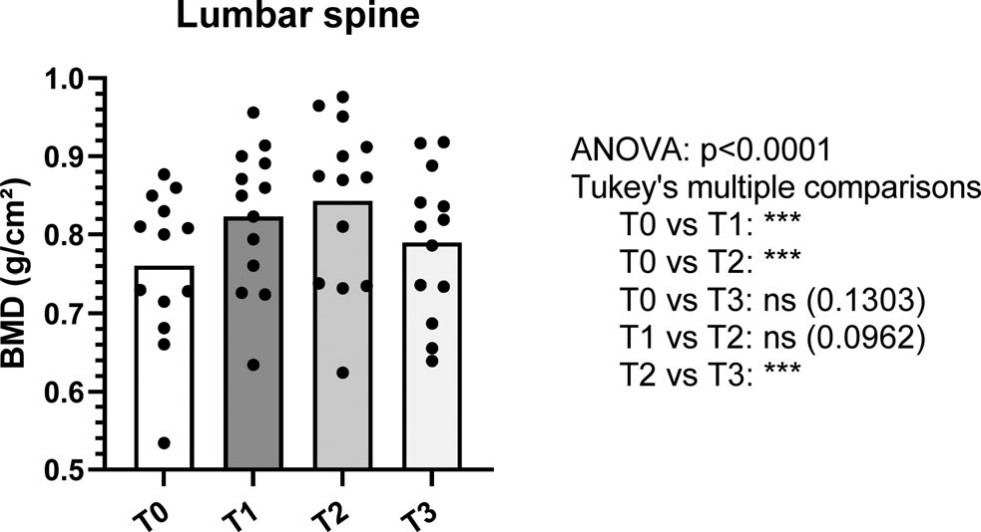
Evolution of bone mineral density at the spine. T0: BMD at denosumab initiation; T1: BMD at the last 60 mg denosumab injection; T2: BMD 6 months after the 15 mg injection of denosumab; T3: BMD 12 months after the 15 mg injection of denosumab. BMD = bone mineral density. ***p value ≤0.0001.

**Fig. 3:**
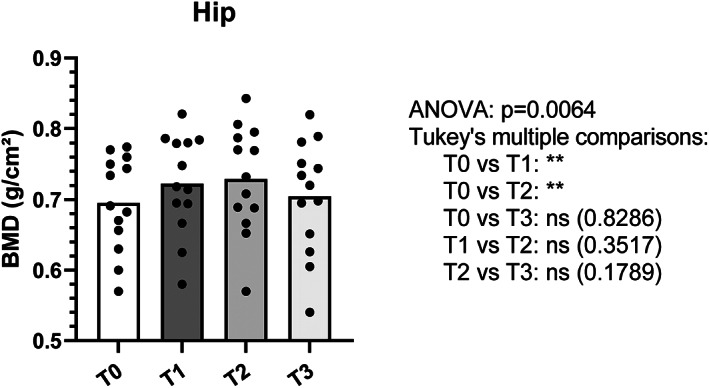
Evolution of bone mineral density at the hip. T0: BMD at denosumab initiation; T1: BMD at the last 60 mg denosumab injection; T2: BMD 6 months after the 15 mg injection of denosumab; T3: BMD 12 months after the 15 mg injection of denosumab. BMD = bone mineral density. **p value <0.01.

The mean gain in BMD during treatment with full doses of Dmab was +7.8% ± 4.4% at the spine and +4.0% ± 3.5% at the hip after 45.2 ± 5 months of treatment (Figs. 2 and 3).

Twelve months after the last 15 mg dose of Dmab (T3) there was a bone loss of −5.4% ± 4.0% at the spine and −3.0% ± 2.0% at the hip (Figs. 2 and 3). In total, 39% of the gain obtained under treatment was preserved at the spine while the bone loss is total at the hip (Figs. 2 and 3).

At T2, 6 months after the last 15 mg dose of Dmab, the CTX median value was 166 pg/mL (95% CI, 33–492) (Table 1). At T3, 12 months after the last 15 mg dose of Dmab, the CTX median value was 549 pg/mL (95% CI, 110–1500). There was no significant correlation between the evolution of BMD and CTX values (Table 1, Fig. S1).

**Table 1:** Evolution of CTX Levels and Bone Loss at the Lumbar Spine Between T2 and T3

Patients	CTX T2	CTX T3	CTX variation (pg/mL)	Spine BMD variation (%)
1	246	426	180	−6.5
2	444	1046	602	−11.2
3	70	331	261	−6.0
4	59	172	113	−4.9
5	100	110	10	−6.9
6	159	1020	861	−9.4
7	492	574	82	−7.8
8	33	179	146	+2.4
9	240	1500	1260	−9.0
10	33	667	634	−6.6
11	151	688	537	−0.5
12	101	177	76	−10.2
13	33	249	216	−4.2

Abbreviation: BMD = bone mineral density; CTX = crosslaps.

One of the thirteen patients had two lumbar vertebral fractures due to a minor strain, 8 months after the last 15 mg dose of Dmab. This patient was 77 years old. For this patient, we reinitiated Dmab.

## Discussion

We proposed our study to patients whose fracture risk seemed low, based on densitometric criteria *T*‐score >−2.5 SD and the absence of fracture during the 2 years preceding the study.

Our study shows that the progressive decrease of Dmab helps preserve close to half of the BMD gain obtained with Dmab to the spine, whereas this strategy is ineffective for the hip.

Our findings of BMD decrease with Dmab doses reduction can be compared to several works studying the efficacy of ZOL post‐Dmab, the results of which are fairly heterogeneous, probably depending on the duration of treatment with Dmab and whether or not the patients had taken bisphosphonates before Dmab. Horne and colleagues^(^ ^8^ ^)^ found in 11 patients that BMD gains obtained during treatment with romosozumab for 1 year and Dmab for 2 years were maintained in 73% (spine) to 87% (hip) of patients, 12 months after receiving an infusion of ZOL (post‐Dmab discontinuation). However, 24 months after ZOL, BMD decreased by −8% at the spine. An observational study including 120 postmenopausal women treated with Dmab for 2–3 years were given a single infusion of ZOL 5 mg, 6 months after the last Dmab injection. Six to Thirty‐six months after ZOL treatment, BMD had decreased significantly by −3.3%, −2.2%, and −1.5%, at the spine, total hip, and femoral neck, respectively.^(^ ^5^ ^)^ Anastasilakis and colleagues^(^ ^9^ ^)^ reported increased lumbar spine BMD 12 months after an infusion of ZOL (+1.7%), but decreasing BMD toward baseline values after 24 months (−0.1%) in the ZOL‐treated women. Recently, Sølling and colleagues^(^ ^4^ ^)^ reported a 2‐year randomized, open label trial, including 59 patients with osteopenia. They administrated ZOL 6 months (6 M group, *n* = 20), or 9 months (9 M group, *n* = 20) after the last denosumab injection or when bone turnover had increased (OBS group, *n* = 21). Their patients had taken Dmab for 4.6 ± 1.6 years, a length of time comparable to our study. The bone losses recorded in their patients are also similar to those of our patients: lumbar spine BMD had decreased significantly by −4.8% ± 0.7%, −4.1% ± 1.1%, and −4.7% ± 1.2% 12 months after ZOL in the 6 M, 9 M, and OBS groups. The progressive dose reduction of Dmab helps preserve close to half of the BMD gain obtained in the spine, but is not effective for the hip.

It should be noted, in our study, that an injection of 15 mg of Dmab is sufficient to prevent bone loss and maintains CTX at a low level in most patients. These findings are in line with a previous report assessing various Dmab regimen including 14 mg of Dmab every 6 months in postmenopausal osteoporosis.^(^ ^10^ ^)^ This dose increased lumbar spine and hip BMD, when compared to placebo, but to a lesser extent than higher doses. However, the 14‐mg dose was not able to induce a sustained reduction of CTX levels over the 6 months of the inter‐dose period.

The limits of our pilot study are linked to the small number of patients and absence of a control group. The main input for the scope is the fact that it is the only prospective study on the effect of the progressive decrease of Dmab. Moreover, we selected patients with high expected risk of bone loss on discontinuation of denosumab because they had no treatment with bisphosphonates before Dmab, and Dmab duration was longer than 36 months.

## Conclusion

To date, no therapeutic protocol using bisphosphonates fully preserves bone gain after Dmab withdrawal. A progressive decrease in treatment preserves ~40% of the gain at the spine. More studies about Dmab tapering are needed and potential usefulness of combining bisphosphonates with low‐dose Dmab remains an unexplored area.

Administration of 15 mg of Dmab is sufficient to prevent complete BMD loss and to keep CTX levels low. This approach could be an alternative when bisphosphonates are contraindicated following Dmab.

## Author Contributions

**Michel Laroche:** Conceptualization; data curation; formal analysis; investigation; methodology; supervision; validation; writing – original draft; writing – review and editing. **Guillaume Couture:** Writing – review and editing. **Yannick Degboé:** Formal analysis; writing – review and editing.

## Disclosures

The authors declare no conflicts of interest.

### Peer Review

The peer review history for this article is available at https://www.webofscience.com/api/gateway/wos/peer-review/10.1002/jbm4.10731.

## Supporting information


**Fig. S1.** Correlation between crosslaps and bone mineral density variations from T2 to T3.

## Data Availability

The data that supports the findings of this study is available from the corresponding author upon reasonable request.
