# Supplementary material for: Discontinuation of Denosumab: Gradual Decrease in Doses Preserves Half of the Bone Mineral Density Gain at the Lumbar Spine
Source: JBMR Plus. 2023 May 18;7(7):e10731. doi: 10.1002/jbm4.10731 (PMC10339081; doi:10.1002/jbm4.10731)
Supplement: Supplementary file 1 — Fig. S1. Correlation between crosslaps and bone mineral density variations from T2 to T3. [file JBM4-7-e10731-s001.docx]

**Supplementary Material**

Supplementary Figure 1: Correlation between crosslaps and bone mineral density variations from T2 to T3

*CTX: crosslaps; BMD: bone mineral density. R²: Pearson R squared for the assessment of the correlation between variations of spine BMD and CTX from T2 (6 months after the 15 mg injection of Denosumab) to T3 (12 months after the 15 mg injection of Denosumab).*
